# The future of intensive care: delirium should no longer be an issue

**DOI:** 10.1186/s13054-022-04077-y

**Published:** 2022-07-05

**Authors:** Katarzyna Kotfis, Irene van Diem-Zaal, Shawniqua Williams Roberson, Marek Sietnicki, Mark van den Boogaard, Yahya Shehabi, E. Wesley Ely

**Affiliations:** 1 Department of Anesthesiology, Intensive Therapy and Acute Intoxications, Pomeranian Medical University in Szczecin, Szczecin, Poland; 2 Department of Intensive Care, Radboud University Medical Center, Radboud Institute for Health Sciences, Nijmegen, The Netherlands; 3 Department of Intensive Care Medicine, University Medical Center Utrecht, Utrecht, The Netherlands; 4 Critical Illness, Brain Dysfunction, and Survivorship (CIBS) Center, Center for Health Services Research, Nashville, TN USA; 5 Department of Neurology, Vanderbilt University Medical Center, Nashville, TN USA; 6 Department of Biomedical Engineering, Vanderbilt University, Nashville, TN USA; 7 Department of Architecture, West Pomeranian University of Technology in Szczecin, Szczecin, Poland; 8 Monash Health School of Clinical Sciences, Monash University, Melbourne, VIC Australia; 9 School of Clinical Medicine, University of New South Wales, Sydney, NSW Australia; 10 Division of Allergy, Department of Medicine, Pulmonary, and Critical Care Medicine, Vanderbilt University Medical Center, Nashville, TN USA; 11 Geriatric Research, Education and Clinical Center (GRECC) Service, Nashville Veterans Affairs Medical Center, Tennessee Valley Healthcare System, Nashville, TN USA

**Keywords:** Outcome, Intensive care unit, PICS, PICS-F, ICU design, Architecture, Neuroesthetics

## Abstract

In the ideal intensive care unit (ICU) of the future, all patients are free from delirium, a syndrome of brain dysfunction frequently observed in critical illness and associated with worse ICU-related outcomes and long-term cognitive impairment. Although screening for delirium requires limited time and effort, this devastating disorder remains underestimated during routine ICU care. The COVID-19 pandemic brought a catastrophic reduction in delirium monitoring, prevention, and patient care due to organizational issues, lack of personnel, increased use of benzodiazepines and restricted family visitation. These limitations led to increases in delirium incidence, a situation that should never be repeated. Good sedation practices should be complemented by novel ICU design and connectivity, which will facilitate non-pharmacological sedation, anxiolysis and comfort that can be supplemented by balanced pharmacological interventions when necessary. Improvements in the ICU sound, light control, floor planning, and room arrangement can facilitate a healing environment that minimizes stressors and aids delirium prevention and management. The fundamental prerequisite to realize the delirium-free ICU, is an awake non-sedated, pain-free comfortable patient whose management follows the A to F (A–F) bundle. Moreover, the bundle should be expanded with three additional letters, incorporating humanitarian care: gaining (G) insight into patient needs, delivering holistic care with a ‘home-like’ (H) environment, and redefining ICU architectural design (I). Above all, the delirium-free world relies upon people, with personal challenges for critical care teams to optimize design, environmental factors, management, time spent with the patient and family and to humanize ICU care.

## Introduction

Delirium is an acute disturbance in attention and awareness with additional disturbances in cognition [1]. Hyperactive delirium may manifest as a combative patient who does not follow the rules of treatment, while hypoactive delirium may manifest as a somnolent patient who is disengaged and inattentive. Delirium may be a prodromal symptom of deranged homeostasis and an early sign of infection or hypoxia. The COVID-19 pandemic brought a catastrophic reduction in delirium monitoring, prevention, and patient care due to organizational issues, lack of personnel, increased use of benzodiazepines and restricted family visitation [2]. These limitations led to increases in delirium incidence, a situation that should never be repeated [3]. The direct result was a world full of deeply sedated, lightly monitored patients, cared for in inadequately staffed ICUs where delirium monitoring and prevention became a very low priority [4].

In the ideal intensive care unit (ICU) of the future, the incidence of delirium will have declined from current levels of approximately 30% [5, 6] to near zero. The fundamental prerequisite to realize this delirium-free ICU is an awake, non-sedated, pain free, comfortable patient. To accomplish this, the future of ICU care will see consistent implementation of standard-of-care interventions to prevent and early detect delirium, founded in the well-established A to F bundle [7–10]. We envision expansion of this bundle to include additional practices that may decrease incidence and duration of delirium. Optimal sedation practices should be applied consistently. New and emerging technologies should be implemented and validated for continuous delirium monitoring. These advances will be facilitated by an innovative architectural design of the ICU environment that optimizes patient comfort, promotes anxiolysis and facilitates holistic, personalized care. These structural and operational changes will provide a strong framework for delirium care in the ICU that will be resilient to challenges such as those arising from the COVID-19 pandemic [2–4]. In this paper we discuss the current burden of ICU delirium and our recommendations and predictions for patient management, environmental changes and infrastructure adaptations that will lead to a delirium-free ICU.

## The burden and long-term consequences of ICU delirium

Delirium undermines the cognitive reasoning itself, challenging Descartes’ *“Cogito Ergo Sum*” (I think, therefore I am), leaving patients vulnerable and potentially forever changed. The experience of delirium is very distressing both for the patient and for the family [11]. Already in ancient times, Hippocrates recognized delirium in severely ill patients as a bad omen [12]. Patients with delirium spend more time mechanically ventilated, more time in the ICU and more time in the hospital with consequently increased health care costs [6, 13–16]. Delirium is associated with increased mortality in the ICU, among frail patients in the hospital [17] and among those with mixed delirium at 90 days [18–20], though an association between delirium and mortality is less apparent when adjusting for disease severity in the ICU [21–23]. As ICU survivorship grows, long-term sequelae of ICU delirium become clearer on long-term functional disability and poor mental health including anxiety, depression, and post-traumatic stress disorder (PTSD). Patients who had delirium more often report problems in activities of daily living and worse scores on sensorimotor function tests at long-term follow-up [24]. New onset cognitive impairment months after ICU discharge is more frequent among patients who suffered delirium during their ICU stay [14, 23, 25, 26], even when adjusting for severity of illness. Anxiety and depression are related to delirium in non-ICU patients [27], although this relationship is less apparent in ICU survivors [28]. PTSD at 1-year after ICU discharge may also be related to delirium, yet this relationship is still inconsistent in current literature [29].

## Overcoming sedation challenges

Good sedation practice in the future should be complemented by a rethink of design and connectivity of the future ICU to facilitate optimal sedation, anxiolysis and comfort using non-pharmacological means supplemented by balanced pharmacological interventions when necessary. This will represent an evolution from the current landscape, where sedation practices are determined by clinicians’ experience, training and individual preferences, institution and ICU case mix, level of teaching, research and education, and health economics in individual countries [30]. The COVID pandemic highlighted these observations and presented new realities, specific to ICU sedation and delirium management [2, 4, 31]. The pandemic has been characterized by deeper sedation, prolonged neuromuscular blockade and immobility, and restricted access to physical rehabilitation and family support, in isolated artificial environment with caregivers in full protective equipment aggravating anxiety, distress and delirium. This has highlighted the fact that there is significant practice variation. Sedative choices are considered as ancillary interventions with little impact on patients centered outcomes, thus critical thinking for the choice of sedative agents and/or sedation depth is currently lacking.

The implementation of the A–F bundle along with the expansion to A–I bundle is pivotal to achieve the goal of standardized, best practices for sedation. As clinicians gain (G) insight into patient needs, transform to holistic and personalized care with ‘home-like’ aspects (H) of the environment and redefine ICU architectural design (I) to optimize multidimensional humanitarian care, optimal sedation practices will take a place of importance in clinical care.

Albeit many limitations, recent sedation trials focused at large on pharmacological interventions and did not show a superiority of one agent over another [32–34]. Nonetheless, current clinical practice guidelines on pain, agitation/sedation, delirium, immobility, and sleep [11], conditionally recommended non-benzodiazepine sedation in ventilated critically ill adults [35] as there are signals that benzodiazepines are associated with increase of delirium onset [25, 36]. Some of these trials, however, demonstrated significant heterogeneity of treatment in older vs younger patients and operative vs medical admission [37]. While the implication of this heterogeneity is yet to be evaluated, it takes us further into individual and personal approach to sedation management. Furthermore, multiple sedative agents have been used in combinations, in most patients. Thus, multimodal sedation should be used to allow easy titration towards light and optimal sedation, and to reduce the adverse events of individual agents. Timely introduction of specific agents that may promote weaning, reduce agitation and delirium will facilitate early weaning and liberation from mechanical ventilation [38]. Moreover, new insights in relation of sedation with personalized care may be provided by trials evaluating the efficacy of patient-controlled sedation to manage symptoms associated with the distress induced by mechanical ventilation [39]. Sedation trials in the future need to incorporate non-pharmacological interventions as part of integrated approach to optimal sedation, anxiolysis and delirium management. Facilitated by the futuristic design of a modern ICU, virtual reality, music therapy, and distraction techniques could all substantially reduce reliance on chemical agents for analgesia, anxiolysis and stress reduction.

## The future of delirium-free ICU design

**Fig. 1:**
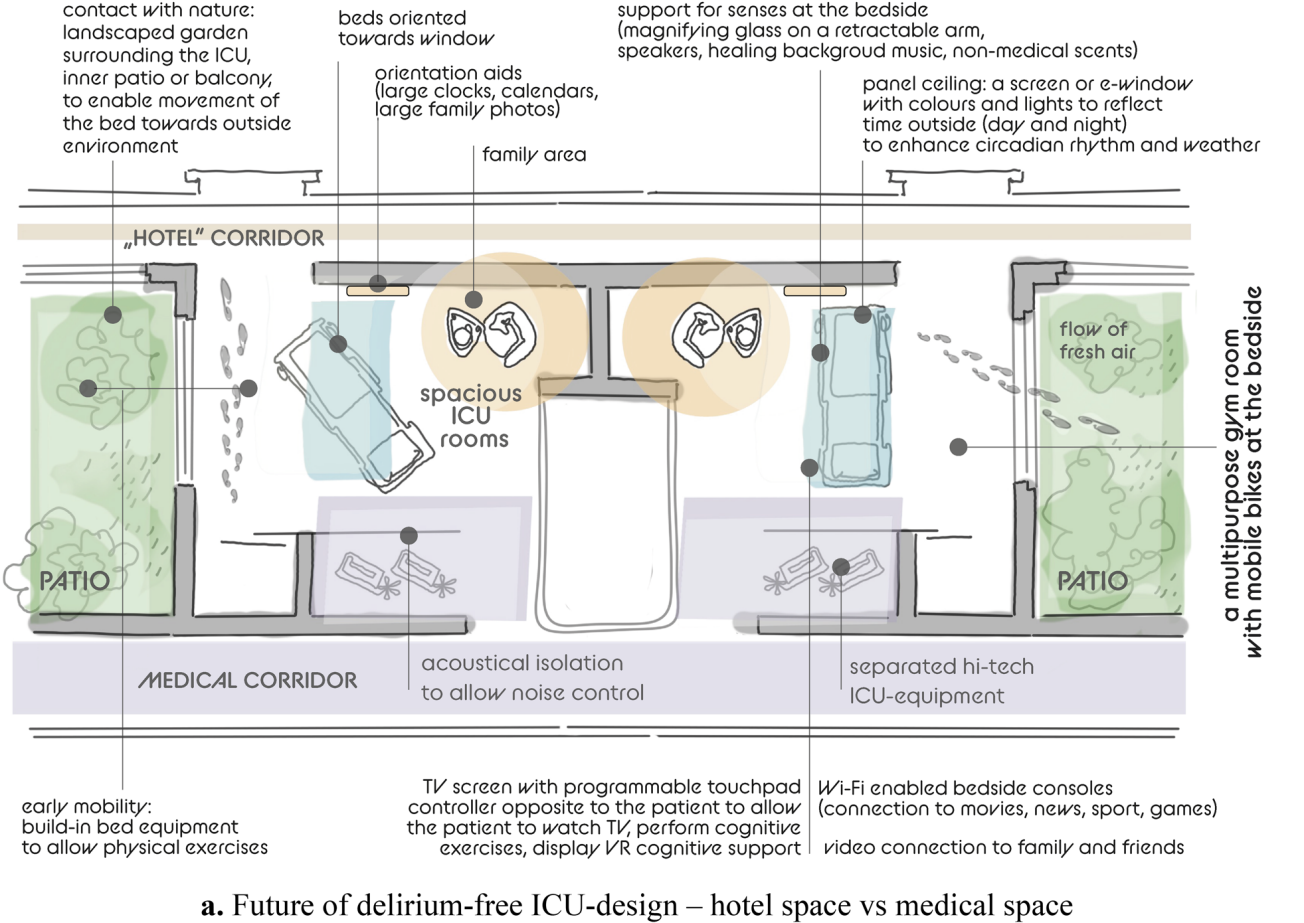

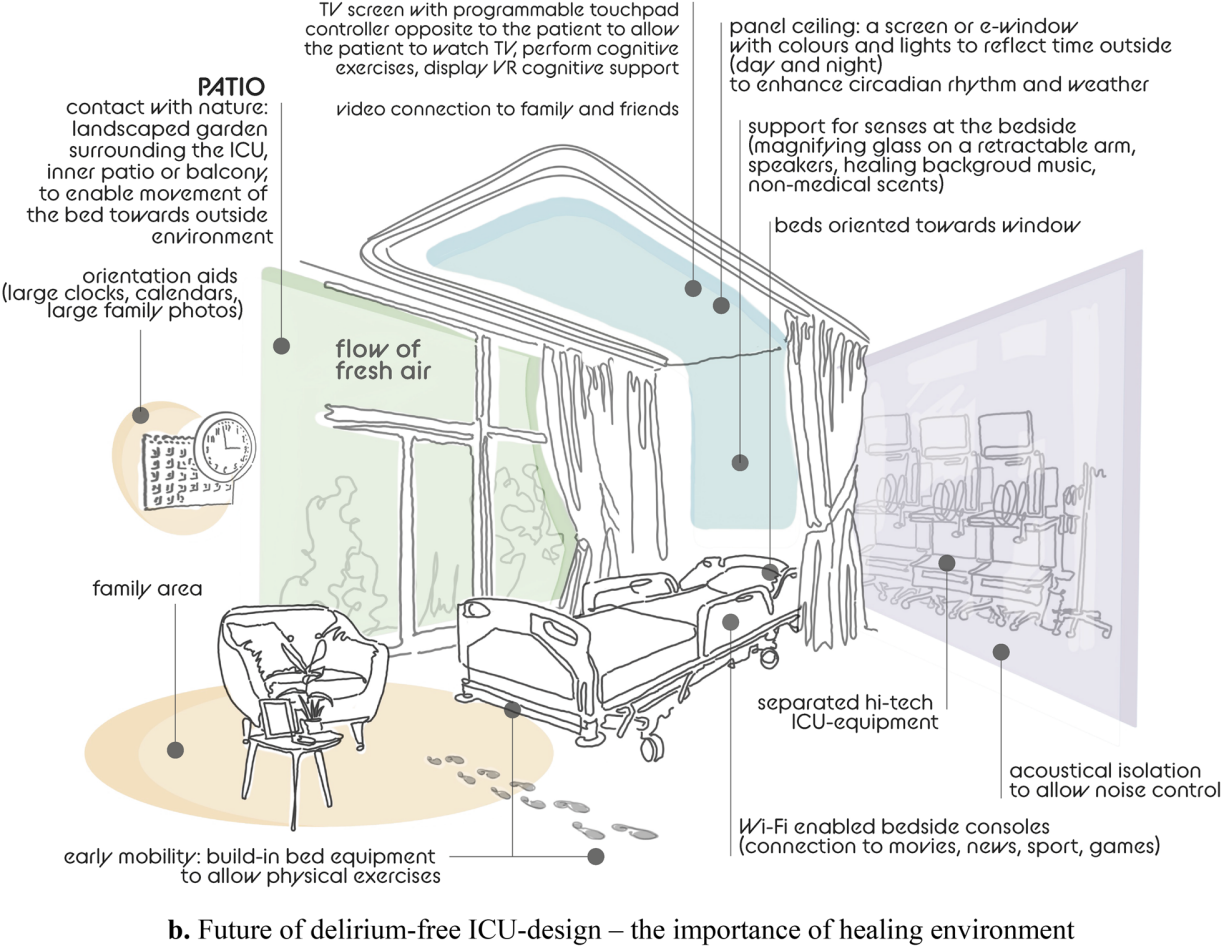
**a** Future of delirium-free ICU-design – hotel space vs medical space. **b** Future of delirium-free ICU-design—the importance of healing environment

Advanced ICU design, turning the highly specialized ICU into “a five-star hotel” with spacious, ergonomic ICU rooms and topographic separation between the medical corridor (for medical teams) and a “hotel” corridor (for family and visitors), may be regarded as part of the process of shifting a “hostile” environment into a “home-like” environment through architectural and interior design modifications (Fig. 1a) [40, 41]. It is known that the physical environment affects physiology, psychology, and social behaviors of those who experience it, both patients and staff [42]. Recently, the idea of neuroesthetics has been introduced to improve mental health conditions and art has emerged as brain stimulation therapy [43, 44]. Visual esthetic experiences can influence neuronal activity associated with the reward system buffering stress response. The use of visual esthetic experiences and art-based interventions have been suggested as improvement in mental health in COVID-19 [45]. Art therapy is becoming an important tool in the armory of psychologists working with patients, therefore patients should be able, alone or with families, to express their emotions visually.

While modern ICUs should separate hi-tech environment and noisy alarm systems from patient accommodation, investment in remote, simple, minimally invasive, and reliable monitoring of sedation, anxiety, sleep, pain, and delirium is urgently needed. The presence of advanced neuromonitoring will allow better management of anxiety, pain, agitation, sleep, and delirium prevention. This rethinking of the ICU outline and equipment use, as well as maximizing the hotel services for patients and families is part of the improvement process to introduce a healing environment minimizing environmental stressors and to aid delirium prevention and management [46–50]. The suggestions for healing environment include sound, light control, floor planning, and room arrangement [51, 52]:

- High-tech medical screen: separation between high-tech ICU-equipment, including alarms, monitors, and patient surroundings to allow noise control acoustically isolated;
- Natural light: beds oriented towards the window, natural windows and/or e-windows, normal use of ambient lights to enhance circadian rhythm [51, 53–55];
- Contact with nature: landscaped garden surrounding the ICU, inner patio or balcony, to enable movement of the bed towards outside environment, flow of fresh air, hydroponic plants (or regular plants, in a glass case);
- Panel ceiling: a screen or e-window: colors and lights to reflect time outside (day and night) to enhance circadian rhythm, clouds and nature [52];
- TV screen with programmable touchpad controller opposite to the patient (separate from the overhead screen) to allow the patient to watch TV, perform cognitive exercises, display VR cognitive support, systems to teach patients about their medical condition,
- Video connection to family and friends, systems to connect patients with similar medical issues, virtual assistant, VR activities to connect with home;
- Wi-Fi enabled bedside consoles with connection to movies, news, sport, games
- Orientation aids: large clocks and calendars, large picture frames for family photographs (avoid hallucinogenic pictures);
- Support for senses: vision—glasses, a magnifying glass on a retractable arm at bedside; touch—allowing tactile stimulation from relatives (touch, embrace), speakers, hearing—healing background music, reduction of noises; smell—allowing non-medical scents into the bedside area;
- Early mobility: build-in bed equipment to allow physical exercises, indoors and outdoors, a multipurpose gym room with mobile bikes at the bedside.

Moreover, a dedicated family area should be provided with comfortable armchair, table, storage cabinet, a video panel that would allow easy, one-touch dialing to reach key family members, integrated speakers so family members visiting can play patient's favorite music from their smartphones among many other ideas (Fig. 1b).

## Patient and family centered care

The presence of the family and loved ones at patient bedside is crucial for healing, so allowing extension of visiting times to 24 h per day, 7 days a week is a quality measurement for the ICU [56]. This means not only that a member of the family can sleep in the same room, bring in children, friends, or pets, but could also play a role in taking care of the patient; family participating. Importantly, the family but also would need psychological and social support to learn how to provide support for the patient [57]. The effectiveness of addressing family needs of critically ill patients involves support groups in and out of ICU, structured communication and/or education programs, providing information brochures to meet family needs or the use of diaries [58]. Nurse-led interventions for improving family outcomes in the ICU include educational interventions with digital storytelling, bundled approach, informational nursing interventions, and nurse-driven emotional support [59]. All these interventions help promote family involvement in their loved one's care and facilitate their decision-making capacity, improving clinician and family interaction, comprehension of the patient’s condition and reduce the development of PTSD. Family satisfaction may be increased with the provision of comfortable physical environments with noise reduction measures [58].

## The importance of coordinated care: expanding the A–F to the A–I bundle

As delirium has significant negative sequelae, the ICU teams of the future will have a strong and consistent focus on its prevention, early recognition, and management. Since the genesis of delirium is multifactorial, interventions will be multidimensional. Removing and treating the underlying cause of delirium is the first and best treatment for delirium. Triggers and drivers of delirium will be managed early and effectively as they are at large preventable and often iatrogenic. Early identification of these triggers with the use of decision-trees might be helpful [60, 61] and will be commonly implemented in electronic health records, to facilitate integration to routine clinical decision-making. Education regarding ICU delirium, including screening for and potential elimination of modifiable risk factors, will be expanded outside of ICU and include all hospital and ambulatory multidisciplinary teams (i.e., surgeons, emergency room physicians, general practitioners, inpatient nursing staff) and even lay people. By doing so we will increase awareness of care practices that may contribute to delirium, decreasing its incidence. This education will also serve to increase recognition of delirium beyond the walls of the ICU, enabling faster intervention and shorter duration of delirium.

The prevention of delirium will hinge on implementing non-pharmacological interventions, which have shown the most potential for success [62–64]. Yet, pharmacological interventions will be useful to manage conditions that can contribute to delirium. Therefore, a delirium-free comfortable patient will mandate a fine balance of pharmacological and non-pharmacological management of pain, anxiolysis, and restorative sleep among other important modalities such as family engagement. The use of a structured framework, to guide ICU nurses and physicians to deliver a combined but balanced pharmacological and non-pharmacological intervention is imperative. Observational studies of compliance and its association with improved outcomes suggest that a bundle, based on the Awakening and Breathing Trial [65] with daily interruption of sedation and spontaneous breathing trials, is a useful framework. The bundle has been expanded over time to its present form; ABCDEF or A–F bundle supported by international practice guidelines [66]. The A–F bundle (with pain, sedation, and delirium management, awaking and breathing trials, early mobilization, and family engagement and empowerment) is therefore a multicomponent and multimodality framework. Importantly, all parts are closely connected with each other, e.g., the choice of sedation and analgesics will likely affect choice of pain assessment, but also success of the awakening trial. The choice of sedation could also affect, and even hinder early mobilization [67], and the occurrence of delirium, particularly when using benzodiazepines [36, 68]. Performing all parts of the bundle, including restricting the use of physical restraints, will be crucial for optimizing patient outcomes, especially regarding delirium prevention.

**Fig. 2:**
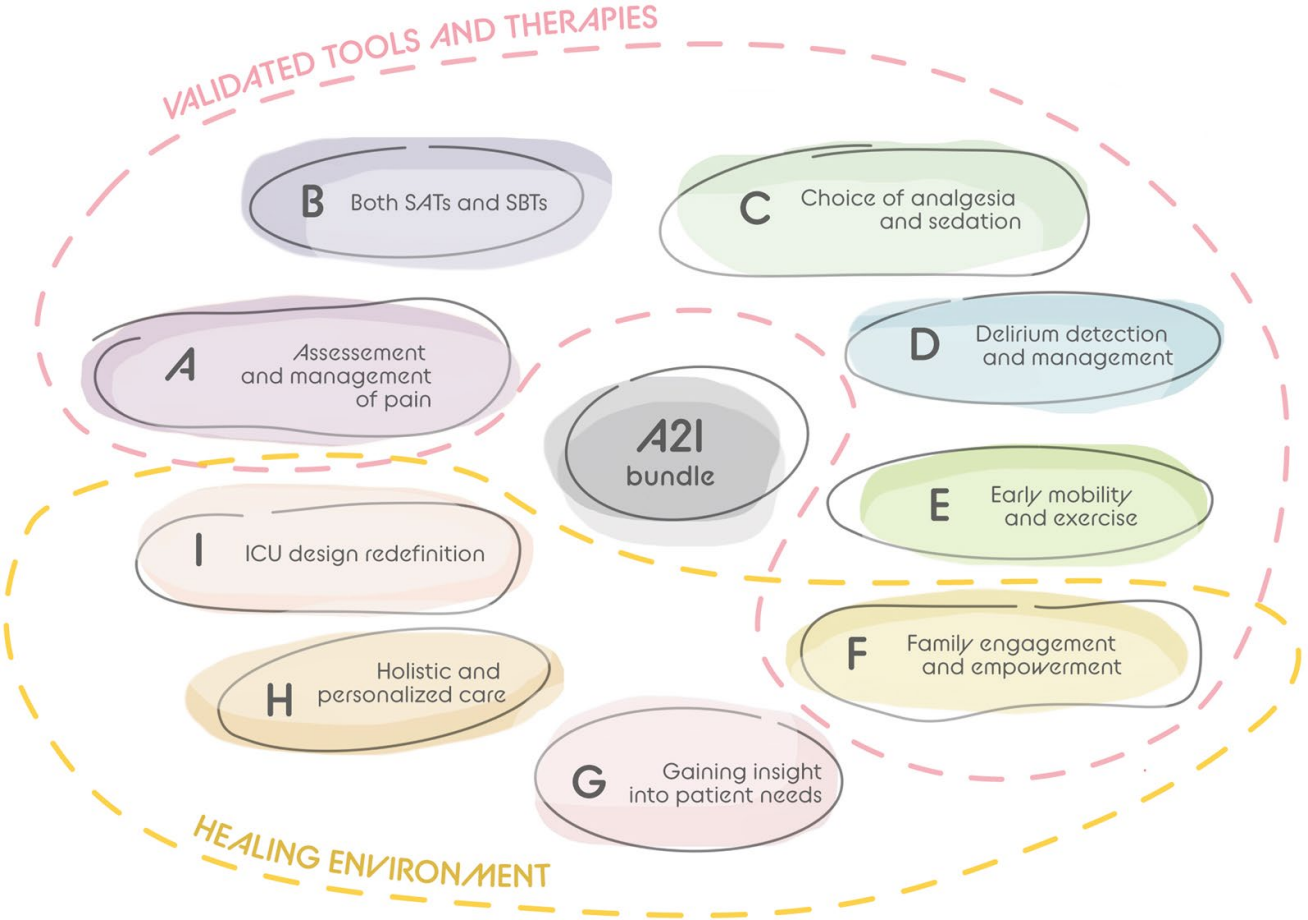
The ABCDEFGHI bundle—A–I bundle. A—Assessment and management of pain: subjective (NRS, VAS) behavioral tools (CPOT, BPS) should be complemented by novel pain assessment technology (ANI, NOL, PPI), multimodal approach to pain, pain-free noninvasive monitoring, pain-free blood drawing for labs. B—Both SATs and SBTs: daily, regular spontaneous awakening trials and spontaneous breathing trials to limit analgesia and sedation needs. C—Choice of analgesia and sedation: good sedation practices complemented by a rethink of design and connectivity of ICU to facilitate optimal sedation, anxiolysis and comfort using non-pharmacological means supplemented by balanced pharmacological interventions when necessary. D—Delirium detection and management: traditional validated tools (CAM-ICU or ICDSC) complemented by novel tools (wireless EEG, NIRS, noninvasive brain electrolyte monitoring, video-assisted delirium signs recognition, electrodermal activity measured by wristband devices). E—Early mobility and exercise: tailor made stepwise physical and cognitive activity programs using specially adapted equipment (virtual reality) and easy access to the outside world. F—Family engagement and empowerment: allowing visits 24/7 (including children and pets), family can sleep in the same room, large picture frames for family photographs, video panel to allow easy reach of key family members. G—Gaining insight: acknowledging patients’ personal needs, preferences, and habits (music therapy, colors, scents) for holistic and personalized care. H—Holistic and personalized care with ‘Home-like’ aspects: providing familiar, safe environment within a customized ICU including provision of circadian rhythm and adequate sleep hygiene. I—ICU design redefinition: environment where patient’s feel safe, comfortable, with recognizable things, not overwhelming (separate hi-tech environment and noisy alarm systems from patient accommodation; remote, minimally invasive monitoring, natural light, access to nature, VR aids). Abbreviations: NRS, numeric rating scale; VAS, visual analogue scale; CPOT, critical care pain observation tool; BPS, behavioral pain scale; ANI, analgesia nociception index; NOL, nociception level index; PPI, pupillary pain index; ICU, intensive care unit; EEG, electroencephalography; VR, virtual reality; NIRS, Near Infrared Spectroscopy, CAM-ICU, Cognitive Assessment Method for Intensive Care Unit; ICDSC, Intensive Care Delirium Screening Checklist

The future of intensive care will see consistent implementation of the A–F bundle. This correlates with improved outcome, including more delirium-free days [8, 9], which could be considered as dose–response relation. Therefore, the A–F bundle could be considered as effective in delirium prevention [66] and reducing the delirium burden. Although it seems that many countries have adopted the A–F bundle, compliance rates on the different components varied between the countries [30], so there is still much to gain in many ICUs to further reduce delirium. Furthermore, the A–F bundle could be further expanded with three additional components (Fig. 2):

- ‘G’ represents ‘Gaining insight into patients’ preferences, habits at home and premorbid lifestyle. This knowledge can be used to tailor interventions such as therapeutic music, pleasant visual stimuli, engaging conversation topics and assistive devices to optimize communication.
- ‘H’ represents ‘Holistic and personalized care’. Integrating other non-pharmacological interventions, based on patients’ preferences, like music therapy, customized ICU environment with ‘home-like’ aspects, could further enhance the effectivity of the multimodality treatment (Fig. 1).
- ‘I’ represents a redefined ‘ICU design’ that would mean an architectural challenge (Fig. 2)—an environment in which patients feel safe and comfortable, including recognizable things from home, yet not overwhelming [40].

This change was brought in by the ICU Liberation concept that underlines humanitarian aspects of patient care that should be supplemented by hi-tech supportive therapy ICU teams have access to [46]. With this extension to an A to I bundle (Fig. 2), we encourage ICUs worldwide to adopt a framework which allows a balanced, early, and effective preventive and management strategies to minimize ICU delirium and its burden.

## The importance of multidisciplinary care

The organization of future zero-delirium ICUs should be based on a balanced cooperation of multidisciplinary teams, including physicians, nurses, physiotherapists, clinical pharmacists, psychologists, speech therapists, dieticians, occupational therapy specialists, spiritual or religious support specialists and social workers to accommodate the specific needs of each patient. Multi-dimensional diagnostic and therapeutic approach guarantees comprehensive assessment and integrated plan for treatment and follow-up [69]. The financial resources of each ICU should include the wide range of medical and non-medical professions necessary at the bedside to provide high quality patient care.

Psychologists should be a part of multidisciplinary ICU team and play a key role in assessing and reducing the distress brought by critical illness for patients and families to provide holistic care and improve outcomes. They should attend the ward rounds on daily basis and provide everyday consultation regarding stress, anxiety, sleep and mood disorders, the effects of sedation and delirium [70]. Early intra-ICU psychological intervention is crucial for recovering from stressful experiences, facilitating communication, sometimes resolving family issues, or formulating plans for long-term care [69].

In addition, an important intervention would be to work with a team of dieticians as gut microbiome imbalance or disruption of the gut-brain axis has been associated with the pathomechanism of delirium [71, 72]. Both anesthetics used in general anesthesia and sedatives used in the ICU can change the composition of gut microbiome and contribute to neuroinflammation [73]. The role of the dieticians is not only to provide balanced nutritional support for ICU patients, but also to use evidence-based structured dietary interventions to prevent delirium through intestinal interventions, enhancing the role of gut-brain axis [71] or modulating the tryptophan metabolism pathway proven important in acute brain disorders [74].

## Future delirium monitoring

The ideal future ICU will include processes and technology to facilitate consistent and reliable delirium monitoring. Future advances in delirium monitoring, including the use of artificial intelligence, electrophysiologic and IT solutions, as well as a reliable biomarker will allow seamless recognition of patients at risk of delirium and allow early management. Video-assisted early delirium recognition is a new development that may be useful in enabling ICU clinicians to early intervene and tackle the underlying cause of delirium.

Yet, currently the mainstay of delirium monitoring is the bedside assessment, and there are numerous assessment tools developed for this purpose. The best validated tools include the Confusion Assessment Method for the ICU (CAM-ICU) [75], the Intensive Care Delirium Screening Checklist (ICDSC) [76] and the 4 ‘A’s Test (4AT) [77]. Yet, there are limitations of reliance on bedside assessment for delirium monitoring: staff must be trained for effective implementation, and it adds to a growing list of tasks for already busy nursing teams. This and other challenges have led to high variability in implementation practices [9, 78]. Moreover, interpretation may be unclear in the context of patients with acute (focal) neurologic disease [79].

There are promising emerging technologies that may be able to capitalize on current knowledge about the physiologic changes associated with delirium to provide impartial metrics for delirium monitoring in the ICU of the future including technologies focusing on typical delirium movements and actions [80]. Recordings of brain activity using electroencephalography (EEG) in delirious patients show an abnormal predominance of slow oscillations (delta activity) [81], decreased faster activities [82] and decreased variability in the EEG signal [83]. These findings are associated with worse outcomes (including mortality) at hospital discharge [84] and may also indicate worse long-term cognitive outcomes [85].

Future EEG-based technology for ICU delirium monitoring will see dramatic evolution. This will be a stark contrast from currently available technology, which is impractical for continuous delirium monitoring due to the need for technical expertise to record and interpret full-montage EEGs and the immobilization of the patient’s head for connection to recording equipment. The most widely generalizable quantitative EEG metrics for accurate delirium detection will also require identification. At present, a few commercially available monitors use limited montage, automated EEG processing to detect related types of brain dysfunction [81, 86–88]. Most of these have yet to develop algorithms for automated interpretation of EEG signals that are robust enough to be used for delirium monitoring in ICU. One exception is the DeltaScan monitor, with fair (69%) sensitivity and fair (69%) specificity, meaning that further improvement is necessary [83]. Wireless EEG recording is an emerging technology for seizure monitoring [89]. Its implementation for delirium monitoring in the ICU patient will represent a significant breakthrough in this field.

Other technologies may also find their way into practical use in the ICU of the future. Brain tissue oxygenation, as measured by near infrared spectroscopy (NIRS), is associated with postoperative delirium in older patients after cardiac surgery [90]. Disturbances in cerebral glucose, lactate and pyruvate level can be observed after severe traumatic brain injury [91]. Noninvasive monitoring of these electrolytes may demonstrate changes in delirious patients. Videomicroscopy is a novel technology that can detect dynamic cellular changes in awake humans [92] and may find utility in delirium monitoring. Finally, patterns in electrodermal activity (EDA) can be measured by wristband devices and are an indicator of psychophysiological arousal [93]. Wristband EDA monitors are currently used for seizure detection in epilepsy patients [94] and represent another potential avenue to the future of delirium monitoring.

## Conclusions

So, is it possible to create a future environment and modes of practice in the ICU where delirium will no longer be an issue? The answer is yes. Reliable, innovative assessment tools (artificial intelligence, biomarkers) and good sedation practices should be complemented by novel ICU design and connectivity, which will facilitate non-pharmacological sedation, anxiolysis and comfort that can be supplemented by balanced pharmacological interventions when necessary. Improvements in the ICU sound, light control, floor planning, and room arrangement can facilitate a healing environment that minimizes stressors and aids delirium prevention and management. It is also possible at a cost of strict adherence to the A–F bundle which is just a part of a larger package of interventions, innovations including new technologies to tackle the delirium problem in the ICU rather than centralizing it and with the introduction of three additional letters of humanitarian care – gaining (G) insight into patient needs, holistic care with a ‘home-like’ personalized care (H) and providing healing environment through redefined ICU architectural design and neuroesthetics (I).

Yet, most importantly, the delirium-free world relies upon people. This means personal challenges for critical care teams whose presence and quality time spent with the patient and their family at the bedside to talk, explain, answer questions, and reassure both patient and family cannot be overestimated.

## Data Availability

Not applicable.
