# Correction to: The future of intensive care: delirium should no longer be an issue

**DOI:** 10.1186/s13054-022-04128-4

**Published:** 2022-09-21

**Authors:** Katarzyna Kotfis, Irene van Diem‑Zaal, Shawniqua Williams Roberson, Marek Sietnicki, Mark van den Boogaard, Yahya Shehabi, E. Wesley Ely

**Affiliations:** 1 Department of Anesthesiology, Intensive Therapy and Acute Intoxications, Pomeranian Medical University in Szczecin, Szczecin, Poland; 2 Department of Intensive Care, Radboud University Medical Center, Radboud Institute for Health Sciences, Nijmegen, The Netherlands; 3 Department of Intensive Care Medicine, University Medical Center Utrecht, Utrecht, The Netherlands; 4 Critical Illness, Brain Dysfunction, and Survivorship (CIBS) Center, Center for Health Services Research, Nashville, TN USA; 5 Department of Neurology, Vanderbilt University Medical Center, Nashville, TN USA; 6 Department of Biomedical Engineering, Vanderbilt University, Nashville, TN USA; 7 Department of Architecture, West Pomeranian University of Technology in Szczecin, Szczecin, Poland; 8 Monash Health School of Clinical Sciences, Monash University, Melbourne, VIC Australia; 9 School of Clinical Medicine, University of New South Wales, Sydney, NSW Australia; 10 Division of Allergy, Department of Medicine, Pulmonary, and Critical Care Medicine, Vanderbilt University Medical Center, Nashville, TN USA; 11 Geriatric Research, Education and Clinical Center (GRECC) Service, Nashville Veterans Affairs Medical Center, Tennessee Valley Healthcare System, Nashville, TN USA

## Correction to: Crit Care (2022) 26:200 https://doi.org/10.1186/s13054-022-04077-y

Following publication of the original article [1], the authors identified a citation error in the Future Delirium Monitoring section and the author name Shawniqua Williams Roberson. The family name contained an error.

The updated paragraph 4 in the Future Delirium Monitoring section is given below which now indicates the correct reference number 89 and the reference is included within this correction article.

**Future Delirium Monitoring**

One exception is the DeltaScan monitor, with fair (69%) sensitivity and fair (69%) specificity, meaning that further improvement is necessary [89].

The incorrect author name is: Given Name: Shawniqua Given Name: Williams Family Name: Roberson.

The correct author name is: Given Name: Shawniqua Family Name: Williams Roberson.

The citation and reference and author group has been updated in this correction article and the original article [1] has been corrected.
